# Advances in Research Related to MicroRNA for Diabetic Retinopathy

**DOI:** 10.1155/2024/8520489

**Published:** 2024-02-12

**Authors:** Yahan Luo, Chunxia Li

**Affiliations:** ^1^Shanghai TCM-Integrated Hospital, Shanghai University of TCM, Shanghai, China; ^2^Shanghai University of Traditional Chinese Medicine, Shanghai, China

## Abstract

Diabetic retinopathy (DR) is a severe microvascular complication of diabetes and is one of the primary causes of blindness in the working-age population in Europe and the United States. At present, no cure is available for DR, but early detection and timely intervention can prevent the rapid progression of the disease. Several treatments for DR are known, primarily ophthalmic treatment based on glycemia, blood pressure, and lipid control, which includes laser photocoagulation, glucocorticoids, vitrectomy, and antivascular endothelial growth factor (anti-VEGF) medications. Despite the clinical efficacy of the aforementioned therapies, none of them can entirely shorten the clinical course of DR or reverse retinopathy. MicroRNAs (miRNAs) are vital regulators of gene expression and participate in cell growth, differentiation, development, and apoptosis. MicroRNAs have been shown to play a significant role in DR, particularly in the molecular mechanisms of inflammation, oxidative stress, and neurodegeneration. The aim of this review is to systematically summarize the signaling pathways and molecular mechanisms of miRNAs involved in the occurrence and development of DR, mainly from the pathogenesis of oxidative stress, inflammation, and neovascularization. Meanwhile, this article also discusses the research progress and application of miRNA-specific therapies for DR.

## 1. Introduction

Diabetic retinopathy (DR) is a severe microvascular complication of diabetes and is one of the leading causes of blindness in the working-age population. Globally, DR accounts for 1.4% of cases in adults with moderate or severe visual impairment [1, 2]. The prevalence of diabetes has been rapidly increasing worldwide, with 8.8% of adults affected in 2015 and projected to rise to 10.4% by 2040 [3]. As the prevalence of diabetes increases and people with diabetes live longer, the incidence of DR is also expected to rise, as it is a complication of diabetes. Currently, there is no permanent cure for DR, but the disease can be prevented or managed. Early detection and timely intervention can prevent the majority of cases resulting in visual impairment [4].

Hyperglycemia can cause various changes in the retina, such as increased retinal vascular permeability, loss of vascular endothelial cells and pericytes, thickening of the vascular basement membrane, abnormalities of retinal neurons and glia, tissue ischemia, and the release of various vasoactive substances, ultimately leading to neovascularization [5, 6]. The classical pathways involved in the pathogenesis of DR include activation through the polyol and hexosamine pathways, accumulation of advanced glycation end products (AGEs), and activation of protein kinase C (PKC) [7–9]. Activation of these biochemical pathways can lead to mitochondrial dysfunction, increased oxidative stress, dysfunction of proinflammatory mediators, activation of the renin-angiotensin system, and upregulation of vascular endothelial growth factor (VEGF) expression, ultimately resulting in increased vascular permeability, vascular dysfunction, and neovascularization [10–13].

Patients with DR are usually unable to detect symptoms in the early stages of the disease [14]. As the disease progresses and affects the macular region, patients may begin to experience varying degrees of vision loss [15]. Clinically, DR is classified into nonproliferative diabetic retinopathy (NPDR) and proliferative diabetic retinopathy (PDR) based on its developmental stage and severity [16]. NPDR is the early stage of DR and presents with progressive development. During this period, the blood-retinal barrier (BRB) undergoes significant changes, including increased retinal vascular permeability, thickening of the basement membrane, and selective loss of peripheral cells [17, 18]. The primary physical signs of NPDR range from mild to severe, with small retinal bleeding spots, microangiomas, venous beading, and retinal microvascular abnormalities [19, 20]. DR is considered to have progressed to PDR when one or more of the following three changes occur: neovascularization, vitreous hemorrhage, or preretinal hemorrhage [21–23]. The key to distinguishing NPDR from PDR is the presence or absence of new blood vessels. PDR is characterized by the possibility of neovascularization of the iris (NVI), neovascularization of the disk (NVD), and neovascularization elsewhere (NVE) [19]. The formation of neovascularization is usually accompanied by a surge in inflammatory factors and myofibroblasts [19]. Due to the unstable microenvironment of neovascularization, new blood vessels are fragile, and vascular contents are prone to exudation, leading to vitreous/preretinal hemorrhage and retinal detachment [24, 25].

Several treatments are known for DR, primarily ophthalmic treatments based on glycemia, blood pressure, and lipid control, including laser photocoagulation [26], intravitreal steroid therapy [27, 28], glucocorticoids, vitrectomy [29], and anti-VEGF medications [30]. Laser photocoagulation is the preferred treatment for diabetic macular edema that is not centrally involved [31, 32]. The mainstay of treatment for PDR is total retinal photocoagulation surgery (PRP) [33, 34]. In addition, vitrectomy is the primary surgical intervention for persistent vitreous hemorrhage and retinal detachment that occurs in the later stages of DR [35], while anti-VEGF medications are effective in treating centrally involved diabetic macular edema with the visual loss [36]. Although these therapies are clinically effective, none of them can completely reduce the clinical course of DR or reverse the retinal lesions [8]. Therefore, there is an urgent need for new treatment discoveries. Many researchers have explored new treatment options for DR. New aspects of therapeutic research currently underway include mediators of the angiopoietin signaling axis, immunosuppressants, nonsteroidal anti-inflammatory drugs (NSAIDs), inhibitors of oxidative stress, and inhibitors of viral viscosity (VVIs) [8, 37]. Moreover, diet is also closely related to DR, and diets including fruits and vegetables can have a protective effect on DR [38].

Nutrients and nutritional supplements have also been found to play important roles in DR; for example, vitamins can affect protein glycosylation, oxidative stress, and retinal blood vessels [39]. This also provides a new direction for the treatment of DR in the future. Furthermore, some preclinical studies suggest that DR gene therapy has great potential in the future to provide patients with a better treatment experience through personalized therapy, enabling patients to decrease the frequency of ocular injections or laser treatments, which can better meet patients' needs in terms of effectiveness and sustainability [40].

Several related studies have reported that genetics and other factors may influence DR progression, which may result in dysregulation of related epigenetic mechanisms such as DNA methylation, posttranscriptional modification of histones in chromatin, and the formation of noncoding RNAs, leading to changes in the expression of various genes involved in the DR process, particularly noncoding RNAs [41–44]. miRNAs have been extensively studied as a novel class of small noncoding RNA. A large body of work has shown that aberrantly expressed miRNAs play key roles in the pathogenesis of microvascular complications, such as oxidative stress, apoptosis, inflammation, and angiogenesis, and are believed to play a critical role in the pathogenesis of DR via the aforementioned pathways [45–47]. miRNAs were also found to control insulin synthesis and regulate cytokines to control processes such as immune response, indirectly affecting the occurrence and development of DR [48].

## 2. Structural and Biological Processes of MicroRNAs

MicroRNAs (miRNAs) are a class of small noncoding RNAs, approximately twenty-four nucleotides in length, found in animals, plants, and viruses. They are key posttranscriptional regulators of gene expression. MicroRNAs are involved in the growth, differentiation, development, and apoptosis of the cell body, and a single miRNA can bind to hundreds of different mRNAs and take part in different biological processes by pairing with different bases of targeted mRNA [49].

The biological process of miRNA starts in the nucleus and ends in the cytoplasm. miRNA transcription is initiated by RNA polymerase II, followed by capping, splicing, and polyadenylation, and, finally, the formation of primary miRNA (pri-miRNA) [50]. In the nucleus, the microprocessor complex consisting of the DROSHA ribonuclease and DiGeorge critical region 8 (Dgcr8) double-stranded RNA-binding protein processes pri-miRNA to form hairpin-structured miRNA precursors (pre-miRNA) [51]. The pre-miRNA is formed by shearing and modification of the stem loop structure, which results in a miRNA: the miRNA double strand [52, 53]. With the help of the human ribonuclease Dicer and double-stranded RNA-binding proteins (dsRBPs), the human immunodeficiency virus transactivating response RNA-binding protein (TRBP) or protein activator of PKR (PACT), one of the double strands (guide strand), is selected and combines with Argonaute family proteins to form an RNA-induced silencing complex [54–58]. During which, the other strand (passenger strand) is degraded [59], and the guide strand, which is retained in the miRISC, eventually forms a mature single-stranded miRNA [60–62]. The RNA-induced silencing complex induces the degradation of the target mRNA or suppresses its translation through complementary pairing with the 3′ UTR of its target mRNA molecule. It is important to note that a single miRNA can target hundreds of mRNAs, and a single mRNA can be suppressed by multiple miRNAs [63].

Recent studies have shown that in addition to the above canonical miRNA biogenesis, some miRNA precursors can be processed to produce the abundant two miRNA strands, where passenger strand is not always degraded, and it can be loaded into the Ago 2 protein and contribute to the regulation of mRNA translation [64–66]. For example, Schober et al. [67] found that the precursor miRNA pre-miR-126 produces two mature miRNA chains, miR-126-3p and miR-126-5p. The guide strand miR-126-3p mediates the formation of angiogenesis and anti-inflammatory factor. The passenger chain miR-126-5p maintains endothelial integrity by targeting delta-like 1 homolog (Dlk1), a negative regulator of endothelial cell proliferation. Villain et al. [68] also reported the role of miR-126-5p in endothelial cell survival during the establishment of the retinal vasculature through the regulation of SetD5 and Sema3A. Yang et al. [64] found that transgenic mice expressing miR-17 precursor can produce considerable levels of guide strand miR-17-5p and passenger strand miR-17-3p, while both miRNA-17-3p and miRNA-17-5p can inhibit the expression of TIMP metalloproteinase inhibitor 3 (TIMP3), and the effect of the combination of them is much greater than that of single miRNA. These effects can greatly increase the invasive ability of tumor cells. It has also been found that the guide strand miR-9-5p [69] can promote the proliferation, migration, and invasion of hepatocellular carcinoma cells by targeting ESR1, while the passenger strand miR-9-3p [70] has been proved to inhibit the proliferation and apoptosis of glioma cells through forkhead box G1 (FOXG1).

## 3. MicroRNAs as Biomarkers of DR

Being a class of small noncoding RNAs, approximately 24 nucleotides in length, miRNAs can migrate outside of cells and enter the humoral circulation by binding with other molecules, a process referred to as circulating miRNAs [71]. However, the mechanism by which circulating miRNAs are sorted into exocrine/small extracellular vesicles (sEV) or retained in cells is still largely unknown [72, 73]. Studies have suggested that the sequence or structural features of miRNAs may influence binding affinity and secretion [74]. Koppers-Lalic et al. proposed a miRNA sorting mechanism based on the 3′ terminal structure [75]. They found that 3′-end adenylated miRNA isoforms were mainly enriched in cells, while their 3′-end uridylated miRNA isoforms were overrepresented in exosomes. Further studies have also shown that miRNAs have sorting sequences that determine their sEV secretion or cell retention, which may be related to the existence of sEV export (EXOmotifs) versus cellular retention (CELLmotifs) sequences [73]. This selective release makes it possible to view circulating miRNAs as biomarkers for various diseases.

Studies have shown that approximately 10% of circulating miRNAs enter circulation through exosomes, while the majority of other circulating miRNAs form complexes with RNA binding proteins such as Ago2, NPM1 (nuclear phosphoprotein 1), and high-density lipoprotein (HDL) [76, 77]. Circulating miRNAs have been detected in various bodily fluids, including blood, tear fluid, and vitreous fluid. These miRNAs can be stably present in bodily fluids and serve as biomarkers for several diseases such as cancer, DR, diabetes, and prostate cancer [71, 78–82]. Several studies have demonstrated that miRNAs have high sensitivity and specificity for the diagnosis of DR and can be used as an effective noninvasive biomarker, and the diagnostic effectiveness can be further improved by using a combination of multiple miRNAs [80, 83]. For instance, it has been reported that miR-9–5p and miR-17–3p are downregulated while miR-210 is upregulated in the serum of patients with DR [84, 85]. In the vitreous humor, miR-125a-5p, miR-125b-5p, and miR-204–5p are downregulated, while miR-21–5p, miR-660–5p, miR-142–3p, miR-19a-3p, miR-142–5p, and miR-15a-5p are upregulated [86]. In aqueous humor, miR-200b-3p, miR-199a-3p, and miR-365–3p are downregulated [87]. The following is a summary of the research progress of microRNA as a biomarker of DR in recent years (Table 1).

## 4. MicroRNAs Participate in DR Development by Different Mechanisms

MicroRNAs have been reported to play a regulatory role in various biological processes, including angiogenesis, oxidative stress, immune response, and inflammation. Dysregulation of these processes can lead to the development of DR and other complications, such as choroidal neovascularization [80, 114–116]. Although several studies have investigated the involvement of miRNAs in DR development, their specific mechanisms and target molecules remain largely unknown. Therefore, it is imperative to investigate the underlying signaling pathways and molecular mechanisms through which miRNAs are involved in DR development and progression.

### 4.1. Oxidative Stress Pathways

#### 4.1.1. HIF-1*α* Signaling Pathway

Hypoxia-inducible factor-1*α* (HIF-1*α*) is a crucial transcription factor in the cellular response to hypoxia. It is highly responsive to changes in oxygen concentration in the cellular environment and plays an important role in responding to decreased oxygen levels or hypoxia [117]. Abnormalities in tissue metabolism can arise due to changes in morphology, function, and insufficient oxygen delivery. Under hypoxic conditions, the expression of HIF-1*α* is increased, resulting in the production of reactive oxygen species (ROS). Excessive ROS accumulation can cause mitochondrial damage, apoptosis, inflammation, lipid peroxidation, and structural and functional changes in the retina [6, 118]. Additionally, oxidative stress, which results from excessive ROS production and inhibition of ROS scavenging by antioxidant defense systems, has been reported to be a pathological consequence of several diseases, including diabetes and its complications [119, 120].

Liu et al. [121] reported that miR-135b-5p expression was elevated in retinal tissue and retinal vascular endothelial cells from DR mice, suggesting its involvement in DR pathogenesis. The authors also investigated the effect of miR-135b-5p on HIF-1*α* expression in DR mouse retinas. Their data showed that miR-135b-5p inhibition suppressed HIF-1*α* expression in DR mouse retinal tissues, leading to a reduction in pathological retinal tissue damage, apoptosis, the formation of the lumen, and the activity and migration of retinal DR vascular endothelial cells. The study further revealed that the downregulation of miR-135b-5p promoted the expression of Von Hippel-Lindau (VHL) protein in DR retinal tissue and retinal vascular endothelial cells. The results suggested that the positive regulation of VHL in DR mice in vivo and in vitro was opposite to the negative regulation of miR-135b-5p. In addition, the inhibition of miR-135b-5p or upregulation of VHL played a protective role in DR by suppressing HIF-1*α* expression.

Similarly, Han et al. [91] found that upregulation of miR-203a-3p could inhibit pathological angiogenesis of the retina in PDR by targeting VEGFA and HIF-1*α*. In a murine model of PDR, the authors observed reduced levels of microRNA-203–3p (miR-203–3p). In contrast, upregulation of miR-203a-3p expression decreased the levels of VEGFA and HIF-1*α* in the retinal tissues of the mouse model of PDR. The dual luciferase reporter gene assay showed that miR-203a-3p is specifically bound to the 3′ UTR of VEGFA and HIF-1*α*. The study also found that upregulation of miR-203a-3p expression inhibited HG-induced proliferation, migration, and tube formation in human retinal microvascular endothelial cells (HRMECS).

Guan et al. [122] identified miR-18a-5p as a possible therapeutic target for the treatment of ocular neovascular diseases. The authors found that miR-18a-5p targets fibroblast growth factor 1 (FGF1) and HIF-1*α*. Overexpression of miR-18a-5p in HRMECS led to a significant reduction in the expression levels of FGF1 and HIF-1*α*, thereby inhibiting pathologic neovascularization.

**Figure 1:**
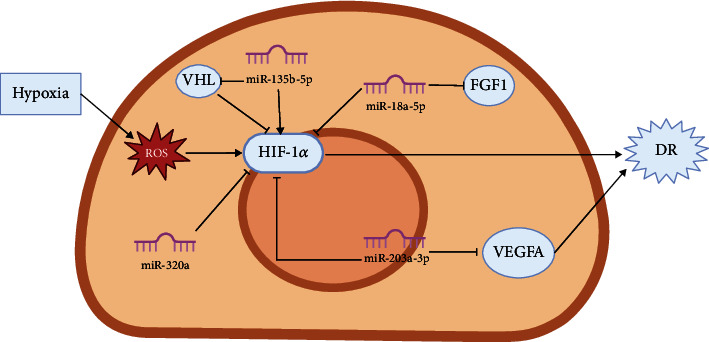
Diagrammatic representation of HIF-1*α*/microRNA mechanism in DR.

Chen et al. [123] found that miR-320a can reduce the damage to Müller cells in DR. The mechanism may be that lncRNA MALAT1 affects the retinal angiogenesis in DR by regulating the miR-320a/HIF-1*α* axis. The results showed that MALAT1 and HIF-1*α* were highly expressed in Müller cells induced by HG, while miR-320a was lowly expressed, and MALAT1 could bind competitively with HIF-1*α*. The overexpression of miR-320a downregulates HIF-1*α* and inhibits the invasion, angiogenesis, and vascular permeability of mouse retinal microvascular endothelial cells (MRMECs). We summarize the miRNAs in HIF-1*α* signaling pathways in DR and present them in Figure 1.

#### 4.1.2. Nrf2 Signaling Pathway

Nuclear factor erythroid 2-related factor 2 (Nrf2) is a crucial participant in preserving mitochondrial homeostasis and structural integrity and acts as a major regulator of cellular redox homeostasis. It plays a significant role in antioxidant stress responses and drug detoxification [124–126]. Activation of Nrf2 and downstream target genes (e.g., superoxide dismutase, glutathione reductase, and glutathione peroxidase) [127] is critical for defending against oxidative stress. An increase in oxidative stress is considered a key metabolic abnormality associated with DR development. Such an increase leads to excessive production of reactive oxygen species (ROS), which damages the inner mitochondrial membrane and results in high levels of superoxide, causing further damage to membrane proteins and disrupting antioxidant defense systems [128, 129]. As mitochondria are the primary sites of ROS generation, they are more susceptible to ROS damage. Damage to mitochondria activates apoptotic mechanisms, leading to accelerated apoptosis of retinal cells and promoting the development of DR [130–132]. Some related studies suggest that Nrf2 may have an important role in maintaining mitochondrial homeostasis in the diabetic retina, thus enabling it to withstand oxidative stress and the development of DR [133–135].

Jadeja et al. [136] conducted correlation expression analysis and demonstrated that miR-144–3p and miR-144–5p target the 3′ UTR of Nrf2 in retinal pigment epithelial cells. They evaluated the expression of miR-144–3p and miR-144–5p in the retina of mice and observed a significant increase in the expression of miR-144–3p in retinal pigment epithelium cells during oxidative stress, while miR-144–5p expression was significantly elevated during the course of oxidative stress. Inhibition of miR-144–3p and miR-144–5p resulted in a significant increase in the expression of Nrf2 and its downstream antioxidant signaling in retinal pigment epithelial cells. The study concluded that miR-144–3p and miR-144–5p have a protective effect on the retina, reducing oxidative stress, and may have important implications for the prevention and treatment of DR.

Luo et al. [137] found decreased expression of Nrf2 and increased expression of miR-93 in blood samples from DR patients and in human retinal pigment epithelial (RPE) cells treated with HG. They demonstrated that overexpression of miR-93 inhibited cellular proliferation and promoted apoptosis, while Nrf2 overexpression abrogated the proapoptotic effect of miR-93, promoted cellular proliferation, and inhibited inflammation. They also found that Nrf2 is a target gene of miR-93 and that miR-93 directly downregulates Nrf2 expression.

Rasoulinejad et al. [138] found that the expression of Nrf2 and miR-146a-5p was significantly reduced in the eye tissues of diabetic rats. They observed a positive correlation between miR-146a-5p and Nrf2 expression and suggested that miR-146a-5p can regulate the expression of Nrf2 as well as inflammation and oxidative stress in diabetic rat eye tissues.

Wang et al. [139] found that overexpression of miR-489–3p impairs the role of transmembrane phosphatase with tensin homology pseudogene 1 (TPTEP1) and that Nrf2 targeted by miR-489–3p is downregulated in human retinal vascular endothelial cells treated with high glucose. They also observed that Nrf2 knockout enhances the impact of miR-489–3p and antagonizes the impact of TPTEP1.

**Figure 2:**
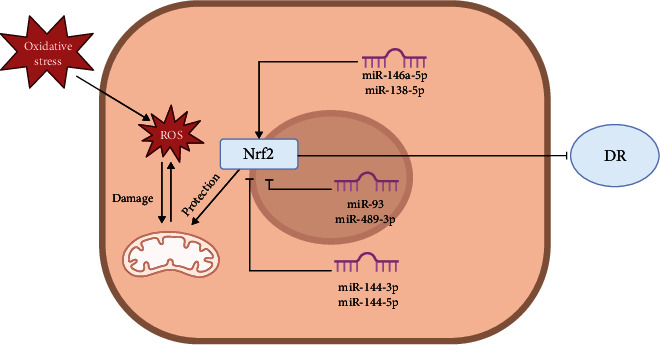
Diagrammatic representation of Nrf2/microRNA mechanism in DR.

Tang et al. [140] found that miR-138-5p can promote the expression of Sirt1 and Nrf2 in the nucleus to alleviate HG damage. Astragaloside IV can increase the expression of miR-138-5p, thereby increasing Sirt1/Nrf2 activity, enhancing cell antioxidant capacity, alleviating cell apoptosis, and inhibiting the progression of DR.  Figure 2 shows the relevant miRNAs in Nrf2 signaling pathways in the progress in DR.

#### 4.1.3. SIRT1 Signaling Pathway

Sirtuin 1 (SIRT1), a highly conserved NAD-dependent deacetylase, is known for its antioxidant and anti-inflammatory properties [141–143]. SIRT1 affects various biological processes, including cellular senescence, apoptosis, lipid metabolism, oxidative stress, and inflammation, by deacetylating histone and nonhistone proteins [144–147]. Due to its ability to reduce apoptosis, inflammation, oxidative stress, and mitochondrial damage, SIRT1 is considered a potential target for the treatment of DR [148–150].

Ji et al. [151] found that miR-34a is upregulated, while SIRT1 expression is decreased in DR rats and retinal tissue cells induced by HG, suggesting that miR-34a and SIRT1 share some common regulation and have a role in DR injury. Overexpression of miR-34a leads to the inhibition of retinal endothelial cell proliferation, while downregulation of SIRT1 can achieve the same effect. These findings suggest that the miR-34a/SIRT1 axis could be a target for DR therapy.

Zeng et al. [90] found that miR-29b-3p was upregulated, and SIRT1 protein was downregulated in DR rats, and subsequent studies showed that in microvascular endothelial cells of the retina, miR-29b-3p was upregulated, while SIRT1 was downregulated. This resulted in an increase in Bax/Bcl-2 expression, which can be reversed by downregulating miR-29b-3p. Therefore, the miR-29b-3p/SIRT1 axis is a potential therapeutic target for DR.

Yang et al. [152] investigated the miR-128–3p and SIRT1 axis and found that in HG-treated retinal microvascular endothelial cells, HG significantly downregulated SIRT1 expression and upregulated miR-128–3p expression, suggesting that SIRT1, a downstream gene of miR-128–3p, has a negative regulatory role, which in turn has an effect on diabetic lesions.

Xiao and Liu [153] found that the expression of miR-217 was upregulated in HG-induced cells, and SIRT1 was discovered to be a direct target of miR-217 by a dual luciferase reporter gene assay. They also discovered that supplementation with miR-217 inhibitors reduced HG-induced cellular injury. This suggests that miR-217 inhibitors would target the SIRT1 gene to reverse the impairment, making miR-217 an important target for DR processing.

Pan et al. [154] found that SIRT1 expression was downregulated in DR- and HG-treated retinal microvascular endothelial cells and that overexpression of SIRT1 reversed proliferation in well-proliferating cells. Although the subsequent studies indicated that SIRT1 positively regulates the expression of miR-20a, miR-20a negatively regulates the activity of the yes-associated protein (YAP)/HIF-1a/VEGFA axis in RMEC. This study also revealed that SIRT1 upregulation could inhibit DR disease progression via the induction of other growth factors by miR-20a.

Shan et al. [155] reported increased expression of miR-195 and BAX and decreased expression of BCL-2 and SIRT1 in retinal cells induced by HG. They also demonstrated a significant targeting relationship between miR-195 and SIRT1, and the knockdown of miR-195 led to increased expression of BCL-2 and SIRT1 and a decrease in apoptosis. These findings suggest that miR-195 is an important driver of DR progression by reducing cellular growth and accelerating apoptosis through the inhibition of SIRT1.

Wang et al. [156] identified a targeting relationship between miR-93–5p and SIRT1 using luciferase reporter gene assay. They found that miR-93–5p is upregulated and SIRT1 is downregulated in rats with DR. Furthermore, expression of SIRT1 was upregulated by the addition of miR-93–5p inhibitors, which led to a reduction in VEGF expression levels and proinflammatory cytokines. These results suggest that miR-93–5p may prevent and treat DR by targeting SIRT1 expression and reducing the inflammatory response to DR.

Wang et al. [157] also found that miR-30b was upregulated and SIRT1 was downregulated in the retina of diabetic mice, indicating a regulatory relationship between miR-30b and SIRT1. Inhibiting miR-30b led to an increase in SIRT1 expression, thereby reducing angiogenesis in retinal microvascular endothelial cells. Additionally, inhibiting miR-30b prevented the progression of PDR in mice by promoting SIRT1 expression.

**Figure 3:**
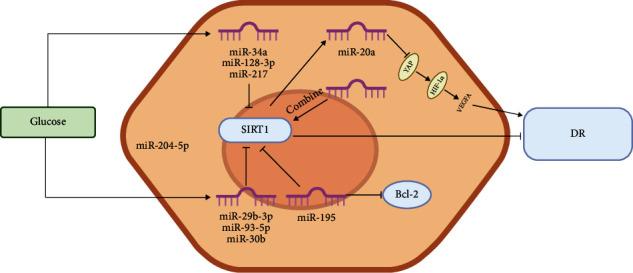
Diagrammatic representation of SIRT1/microRNA mechanism in DR.

Shi et al. [158] found that the expression of circKMT2E in the retina of diabetic mice was more than twice as high as that of miR-204–5p and that circKMT2E plays a role in DR pathogenesis by acting as a sponge for miR-204–5p. They speculated that miR-204–5p can bind to SIRT1 and interact with its target protein to regulate various functions of cells, including the inflammatory response, proliferation, and apoptosis. We have collated the current research on miRNAs in SIRT1 signaling pathways in DR (Figure 3).

#### 4.1.4. AKT Signaling Pathway

The serine/threonine kinase AKT, also known as protein kinase B (PKB), is a proto-oncogene that has three isoforms, AKT1 (PKB*α*), AKT2 (PKB*β*), and AKT3 (Pub), which have markedly different or even opposing functions in cancer and physiology [159–161]. AKT is a key component of the PI3K/AKT signaling pathway, which is activated by the second messenger PI3K and negatively regulated by PTEN. AKT primarily mediates disease development by promoting cell survival and inhibiting apoptosis [162]. Several studies have also demonstrated that the ability of AKT to inhibit apoptosis is dependent on glucose metabolism. Activation of AKT does not inhibit cell death but instead renders cells more susceptible to metabolic stress [163–165]. Since the development of DR is closely linked to the diabetic process, studies have shown that the signaling pathways related to AKT are closely related to oxidative stress and proinflammatory responses in DR [166–168].

Lu et al. [169] found increased expression of miR-21 and AKT-related genes and decreased expression of phosphatase and tensin homolog (PTEN) in DR rats, suggesting a role for miR-21 in DR. They reported that miR-21 can activate signaling pathways related to AKT, and furthermore, miR-21 inhibitors can cause cells to overexpress PTEN, inhibit apoptosis, and promote angiogenesis. However, miR-21 represents an important target for DR therapy.

In order to study its role in the BTG1-mediated AKT pathway, Zhang et al. [92] used rats to establish a DR model with miR-183 mimetic and inhibitors. They found that miR-183 expression was upregulated and BTG1 was decreased in the retinal cells of DR rats. Recent studies have shown that BTG1 is the target gene of miR-183, and inhibition of miR-183 expression could positively regulate BTG1, thereby inhibiting vascular endothelial cell growth in DR rats in vivo through signaling pathways related to AKT. Thus, miR-183 silencing is a target for DR therapy.

Wang et al. [170] found that the expression of miR-199a-3p was decreased but that VEGF expression was increased in HG-induced cells. The miR-199a-3p inhibitor may also cause cellular growth and promote angiogenesis. Upregulation of miR-199a-3p has an opposing effect. In this study, it is reported that miR-199a-3p can inhibit signaling pathways related to AKT, resulting in reduced angiogenesis in DR, which may act through VEGF factors.

Liu et al. [171] noted that Müller glia are the sole source of aberrant expression of miR-9–3p, which activates phosphorylation of VEGFR2. The specific pathway could be accomplished with the binding of miR-9–3p to sphingosine-1-phosphate receptor S1P_1_ through the S1P_1_/AKT/VEGFR2 signaling pathway, opening up new opportunities for potential therapeutic targets for DR and elucidation of mechanisms of disease progression.

**Figure 4:**
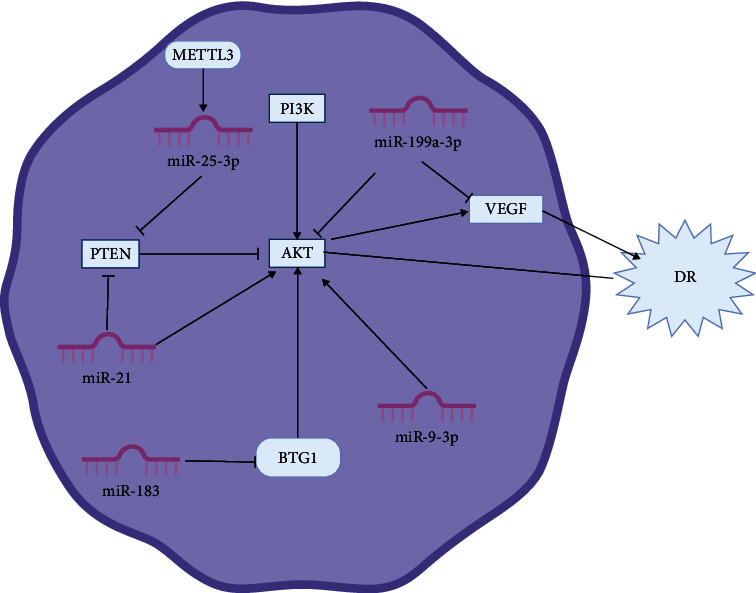
Diagrammatic representation of AKT/microRNA mechanism in DR.

Zha et al. [172] found that high glucose inhibited the expression of METTL3 and miR-25-3p in RPE cells. After upregulating miR-25-3p, the pathological condition of DR improved. In addition, this study suggests that PTEN may be negatively regulated by miR-25-3p, and overexpression of METTL3 increases p-AKT levels by targeting the miR-25-3p/PTEN axis. The upregulation of PTEN consistently hindered the protective effect of METTL3. In summary, METTL3 enhanced the vitality of HG cells by targeting the miR-25-3p/PTEN/AKT axis.  Figure 4 shows the miRNAs in AKT signaling pathways in the progress of DR.

### 4.2. Inflammation-Related Signaling Pathways

#### 4.2.1. NF-*κ*B Signaling Pathway

Nuclear factor-*κ*B (NF-*κ*B) is a family of core dimer transcription factors that coordinate inflammatory responses and can be activated by various stimuli and complex signaling pathway networks [173, 174]. NF-*κ*B is a highly important intracellular nuclear transcription factor and is found in nearly all animal cells. The NF-*κ*B signaling pathway is a typical proinflammatory pathway involved in a variety of biological processes, such as inflammatory and immune responses, cell proliferation, and apoptosis [175, 176]. In related studies, NF-*κ*B has been shown to be associated with apoptosis and oxidative stress in peripapillary retinal cells from DR patients [177–179].

He et al. [180] found that miR-30c-5p inhibited the expression of the PLCG1 target gene, thereby suppressing the activation of the PKC/NF-*κ*B pathway and reducing the inflammatory response in DR. PLCG1 is a member of the PLC family, and its downstream signaling factor, PKC, activates the NF-*κ*B pathway, resulting in the induction of inflammation [181]. Upregulation of miR-30c-5p improved retinal vascular inflammation in DR, as verified by in vivo experiments.

Shi et al. [182] discovered that DR rats exhibit a significant reduction in miR-26a-5p expression levels compared to normal rats. The injection of miR-26a-5p analogs resulted in the attenuation of retinal nerve layer thickness and ganglion cell count reductions in DR mice. These findings suggest that miR-26a-5p plays a role in DR prevention. Additionally, the study demonstrated that miR-26a-5p could decrease the expression of NF-*κ*B in DR rats by negatively regulating PTEN expression and reducing inflammation-induced damage.

For example, Xu et al. [183] found that miR-18b expression was significantly reduced in DR rat retina, and inhibition of miR-18b expression was shown to promote human retinal microvascular endothelial cell proliferation in high-glucose culture. The authors found that miR-18b dampened the inflammatory response by activating MAP3K1 and inhibiting the phosphorylation of NF-*κ*B, which in turn slowed the progression of DR.

Rasoulinejad et al. [138] observed increased expression of NF-*κ*B and decreased expression of miR-146a-5p in DR rat eye tissues. This study suggested that miR-146a-5p might be involved in the preventative therapeutic mechanism of DR. It was also established that miR-146a could inhibit NF-*κ*B-mediated activation of inflammation through the suppression of the expression of its target genes, such as IRAK1 and TRAF6. The progression of DR could also be prevented by increasing miR-146a expression to enhance endothelial function.

**Figure 5:**
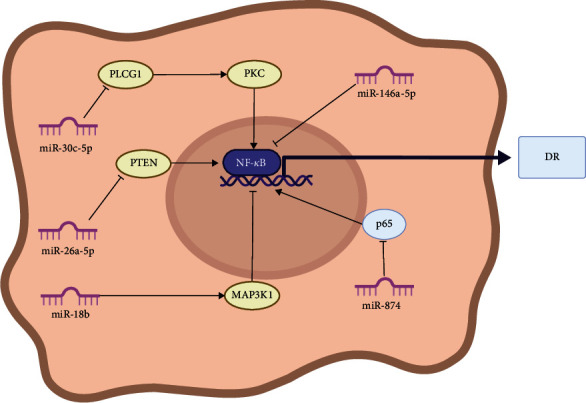
Diagrammatic representation of NF-*κ*B/microRNA mechanism in DR.

Li et al. [184] found that miR-874 can target the degradation of p65. This study suggests that miR-874 regulates NF-*κ*B signaling pathway by targeting p65 for degradation to further improve DR. Compared with normal rats, DR rats showed a decrease in miR-874 expression, an increase in VEGF and Ang II protein expression, a decrease in capillary pericytes, an increase in vascular endothelial cells, and an increase in the p65 expression in the retina. These changes were better in diabetic rats injected with miR-874 mimetics but worsened in diabetic rats injected with miR-874 inhibitors. We summarize the current studies of NF-*κ*B signaling pathways in DR and categorize the relevant miRNAs in Figure 5.

#### 4.2.2. MAPK Signaling Pathway

The mitogen-activated protein kinase (MAPK) signaling pathway is a ubiquitous signaling cascade in eukaryotic cells that regulates various physiological functions, including cell proliferation, differentiation, apoptosis, and stress response, and its dysregulation has been linked to cancer development [185, 186]. The MAPKs are a family of nuclear-expressed kinases that phosphorylate downstream targets to regulate gene expression. Major pathways include the c-Jun amino-terminal kinase (JNK), p38MAPK, and extracellular signal-regulated kinase (ERK), which includes extracellular signaling-regulated protein-1/2/3/4/5/7/8, amino-terminal c-Jun 1/2/3, and p38-MAPK (*α*, *β*, *δ*, and *γ*) [187, 188]. In related studies, various extracellular stimuli, such as oxidative stress and inflammation, have been shown to activate the MAPK signaling pathway [189, 190].

Zhang et al. [191] investigated the role of microRNA-141–3p (miR-141–3p) in docking protein 5- (DOK5-) mediated mitogen-activated protein kinase (MAPK) signaling pathway and found that miR-141–3p can activate the MAPK pathway by inhibiting the DOK5 gene. Upregulation of miR-141–3p was found to inhibit the proliferation of retinal vascular epithelial cells, as well as angiogenesis, and promote apoptosis of RGCs.

Cheng et al. [192] observed reduced levels of miR-181b in diabetic mice and showed that overexpression of miR-181b-improved endothelial function inhibited the generation of intravascular reactive oxygen species (ROS) and suppressed vascular inflammation in diabetic mice. The authors also found that miR-181b may be located downstream of the AMP-activated protein kinase (AMPK) signaling pathway, and activation of AMPK was able to upregulate miR-181b levels in endothelial cells. Activation of the AMPK/miR-181b axis was shown to ameliorate impaired endothelial cell function in a high-glucose environment.

#### 4.2.3. TLR4 Signaling Pathway

Toll-like receptor 4 (TLR4) is a member of the toll-like receptor (TLR) family of receptors and is involved in the immune response. Excessive activation of TLR4 initiates the production of various inflammatory factors that are associated with the development of numerous diseases, such as sepsis, endothelial dysfunction, atherosclerosis, diabetes, rheumatoid arthritis, cardiovascular disease, and metabolic syndrome [193–198]. In DR, the progression of the disease is associated with TLR4 signaling pathway activation, and inhibiting the expression of TLR4 signaling pathways may slow down the DR process [199–201].

For instance, Liu et al. [202] found that interferon alpha 2 (IFNA2) was reduced in diabetic rats, and TLR4 signaling pathway activation was associated with the progression of DR lesions in rats. In this study, they showed that IFNA2 is a target gene of miR-499–3p, which downregulates IFNA2. In DR rats, the upregulation of miR-499–3p expression led to a decrease in IFNA2, which led to the activation of TLR4 signaling pathways. This study also found that downregulation of miR-499–3p attenuated DR lesions in the retina.

Li et al. [203] found that miR-486–3p can be induced within mesenchymal stem cells of bone marrow to generate exosomes, and TLR4 is a target of miR-486–3p, suggesting that there is a link between miR-486–3p and DR disease pathology. In this study, they showed that positive regulation of miR-486–3p can downregulate TLR4, thus inhibiting oxidative stress, inflammation, and apoptosis via the TLR4/nuclear transcription factor-*κ*B (NF-*κ*B) axis, thereby inhibiting DR lesion progression.

**Figure 6:**
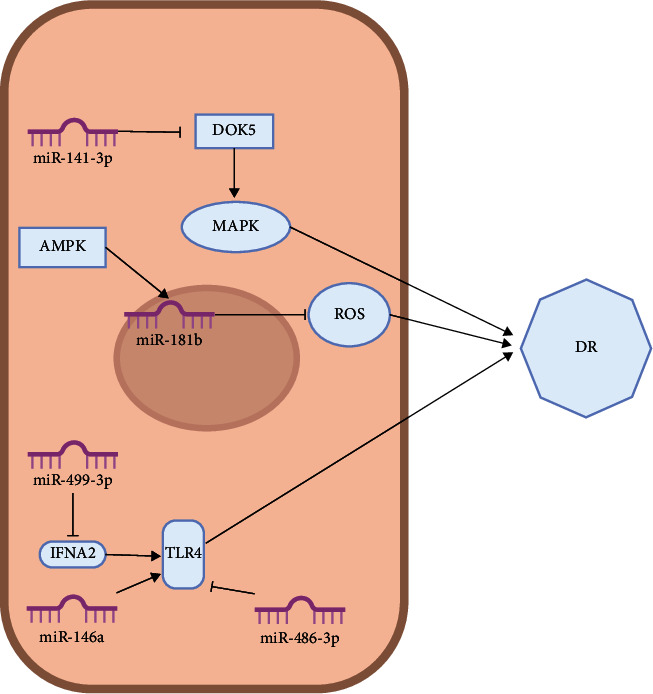
Diagrammatic representation of MAPK/TLR4/microRNA mechanism in DR.

Cao et al. [204] found that the secretion of bone marrow mesenchymal stem cell miR-146a reduced TNF-*α* and the levels of IL-1 and IL-6, suggesting that miR-146a can reduce the inflammatory response in DR mice. Further research has found that miR-146a can reduce the activity of TLR4. In addition, overexpression of TLR4 reverses the effects of miR-146a on the proliferation, apoptosis, and inflammation of microglia. Prompt miR-146a can be adjusted by adjusting toll-like receptor 4 (TLR4)/myeloid differentiation factor 88(MyD88)/nuclear transcription factor-*κ*B (NF-*κ*B), which in turn regulates the inflammatory response pathway of DR. This serves as experimental evidence for the prevention and treatment of DR.  Figure 6 shows the relevant miRNAs in the MAPK signaling pathways and TLR4 signaling pathways in the progress of DR.

### 4.3. Antineovascular Signaling Pathways

Vascular endothelial growth factor (VEGF) is a prominent factor involved in the pathogenesis of DR. Dysregulated production and release of VEGF lead to vascular endothelial cell proliferation and migration, resulting in neovascularization and increased vascular permeability [205]. VEGF comprises several isoforms, including VEGF-A, VEGF-B, VEGF-C, VEGF-D, VEGF-E, VEGF-F, and placental growth factor (PGF) [206]. Among these isoforms, VEGF-A plays a crucial role in regulating angiogenesis [207]. Although anti-VEGF pharmacotherapy has been extensively used in the clinical management of DR, its efficacy remains limited, and adverse side effects are possible. In this review, we summarize the roles of VEGF-related miRNAs in DR with the aim of providing new insights into antineovascular therapy in DR (Table 2).

## 5. MicroRNA Therapy

MicroRNAs are key regulators of cellular signaling cascades, and changes in their expression play a critical role in the alteration of protein expression in many diseases [217]. There are two main strategies for miRNA therapy: inhibition of disease-driving miRNA expression to reduce or block its expression and promotion of the expression of pathologic suppressors of disease [218].

MicroRNAs that drive pathology are often upregulated during the disease process and must be suppressed and returned to normal levels. Five approaches are typically used to inhibit miRNA expression: (i) anti-miRNA oligonucleotides, which are chemically modified single-stranded oligonucleotides complementary to the target miRNA that prevent its interaction with the target gene by binding to the mature miRNA of interest [219, 220]; (ii) miRNA sponges, which are competitive inhibitors with multiple miRNA target binding sites to block the interaction between miRNA and their target mRNA [221]; (iii) small molecule inhibitors, designed using bioinformatics tools or identified by experimental screening of pharmacologically active compounds [222, 223], which function through protein interactions involved in miRNA biogenesis or through inhibition of miRNA-target interactions via binding to specific miRNA secondary structures [221]; (iv) masking of miRNAs, a novel method developed by Xiao et al. consisting of single-stranded 2′-O-methyl-modified antisense oligonucleotides with a high affinity to the predicted miRNA binding site in the 3′ UTR of the target mRNA, rendering the miRNA incapable of binding to the corresponding binding site and thereby inhibiting its target gene (mRNA) [224]; and (v) nucleic acid immobilization using locked nucleic acids (LNA), an oligonucleotide analog with a ring structure formed by the 2′-O atom and the 4′-C atom of ribose linked by a methylene bridge. Locked nucleic acids (LNA) can form a complementary pairing with RNA, single-stranded DNA, and double-stranded DNA with higher affinity [225, 226].

MicroRNAs that act as disease suppressors are often lost during disease development. Promoting the expression of these suppressors can help suppress disease development. MicroRNA mimics are an effective tool for this purpose. These are chemically engineered small double-stranded RNA molecules that mimic endogenous mature miRNA molecules, complementing the alternative processing of the relevant miRNA molecules [227].

## 6. Summary and Discussion

The most effective approach to managing DR is early detection, prevention, and treatment. As many patients are asymptomatic during the early stages of the disease, early detection is crucial [228]. Ophthalmological examinations, such as pupillary dilation and retinal assessment, are currently the available screening modalities [229]. However, early detection and diagnosis of DR still present a challenge worldwide, particularly in middle- and low-income countries. The cost of ophthalmic screening devices and the insufficient number of ophthalmologists hinder the early screening process. Nevertheless, miRNAs can circulate in the blood, providing a novel means of early screening for DR. The identification of a miRNA or group of miRNAs as molecular biological markers for the onset of DR, and the detection of the levels of such markers in the blood, may lead to the early diagnosis of DR and even classification of DR progression by magnitude of grade change, making it a more specific treatment. This has far-reaching implications for DR prevention and treatment in the future.

In recent years, an increasing number of miRNA research reports have been published, and miRNAs have become the focus of attention as both biomarkers and therapeutics. miRNAs can be found stably in serum and other bodily fluids and have been used as biomarkers in numerous diseases due to their endogenous nature, stability, and noninvasive biopsies, such as diabetes, diabetic nephropathy, DR, ophthalmic diseases, cardiovascular diseases, and various cancers [230–236]. However, it should be noted that miRNAs still have many limitations as a biomarker of DR, such as lack of specificity, sample source limitations, and other methodological limitations. miRNAs may have overlapping expression in different diseases, and the variability between different populations and the variability between ethnic groups can limit its guiding characteristics. The abundance of miRNAs in the body fluids of DR patients is low, and most of them can only be detected in blood samples or aqueous humor, which to some extent limits the source of samples. At the same time, the lack of highly efficient clinical collection methods, the immature miRNA isolation and purification techniques, and the lack of standardized procedures ultimately limit its application in clinical practice. Nevertheless, miRNAs still have great potential in the detection of DR, and future research may address these limitations.

miRNAs may also serve as therapeutic agents for various cancers, and their ability to target multiple molecules provides them with a unique advantage over approaches that target single genes [237, 238]. Several miRNA therapies have demonstrated substantial preclinical efficacy for various diseases. For example, miR-10b-5p is in preclinical development and is believed to be useful in the treatment of diabetes and related disorders of gut motility [239]. Preclinical studies have also shown that inhibitors of miR-103/107 improve insulin sensitivity in obese mice [240]. MicroRNA therapeutics have shown great therapeutic promise in clinical settings. However, it still faces many challenges, such as the selection of effective drug delivery routes, controlling the in vivo stability of relevant designer drugs, efficient targeting of specific tissues and cells, determining drug delivery mode and dose, controlling vector-miRNA interaction, and reducing immune responses and side effects.

MicroRNAs have unlimited therapeutic potential and may offer novel therapeutic options for DR. However, it is undeniable that much work needs to be done before miRNA therapeutics can be translated into the clinic. Further research is required to refine miRNA therapeutics.

## Figures and Tables

**Table 1:** Summary of microRNAs as biomarkers of DR.

miRNAs	Changes in DR	Tested tissues	Targets	Cite
miR-21	↑	Endothelial cells	Endothelial cell dysfunction	[88]
miR-216a	↓	Rat retina, human retinal microvascular endothelial cells (HRMECS)	Suppression of nitric oxide synthase 2 (NOS2)/the Janus kinase (JAK)/signal transducer and activator of transcription (STAT)	[89]
miR-29b-3p	↑	HRMECS	Blocking SIRT1	[90]
miR-203a-3p	↓	Rat retina, OIR	VEGFA, hypoxia-inducible factor-1*α* (HIF-1*α*)	[91]
miR-183	↓	Rat retina	PI3K/Akt/VEGF, upregulating BTG1	[92]
miR-200b	↓	Diabetic mouse retina, RMECs	VEGF	[93]
miR-150	↓	Endothelial cells	TNF-*α*, NF-*κ*B	[94]
miR-204	↑	Endothelial cells	SIRT1, Bcl-2	[95]
miR-3197, miR-2116-5p	↑	Retinal endothelial cells (RECs)	Notch homolog 2 (NOTCH2)	[96]
miR-93	↓	Serum	Transforming growth factor-*β* (TGF*β*), VEGFA	[97]
miR-335-3p	↓	Plasma	Endothelial growth factor (EGFR)	[98]
miR-205-5p	↓	HRMEC	Malat1, VEGF	[99]
miR-425-5p	↑	Serum	—	[100]
miR-320a	↓	Plasma	PTEN	[101]
miR-122	↑	Serum	VEGF	[102]
miR-26a-5p	↓	Plasma	PTEN	[103]
miR-431-5p	↑	Serum extracellular vesicles	—	[104]
miR-29c-3p	↓	Plasma	VEGF	[105]
miR-6a-56p, miR-20a-5p, miR-20b, miR-3a-20p	↓	Retina and serum	VEGFA	[106]
miR-3-320p, miR-495b	↑	Plasma and serum	—	[107]
miR-1281	↑	Plasma and serum	VEGFA	[108]
miR-4448, miR-338-3p, miR-485-5p, miR-9-5p	↓	Serum	SIRT, forkhead box O (FOXO) 1, FOXO3	[84]
miR-374a	↑	Serum	—	[109]
miR-214-3p, miR-218-5p, miR145-5P	↓	Tear exosomes	—	[110]
miR-4328, miR-4422, miR-548z, miR-628-5p	↓	Serum	—	[111]
miR-21-3p, miR-30b-5p	↑	Plasma and serum	—	[112]
miR-17-3p, miR-20b	↓	Serum	—	[113]

**Table 2:** Summary of miRNA treatment of DR via anti-VEGF.

miRNA	Expression in DR	Mechanism of action	Regulation of VEGF by miRNA	Pathogenic function	Reference
miR-377–3p	Downregulation in serum exosomes	Lack of it increases VEGF expression	Negative regulation in serum exosomes	Inhibit the development of DR	[208]
miR-15b	Downregulation in serum	miR-15b regulates the expression of VEGF by targeting the 3′ untranslated regions to inhibit its transcription	Negative regulation in serum	Inhibit the development of DR	[115]
miR-409–5p	Upregulation in retinal tissues, in mRMECs, and in vitreous fluid	Its overexpression increases the expression and secretion of VEGF	Positive regulation in retinal tissues, in mRMECs, and in vitreous fluid	Promote the development of DR	[209]
miR-203a-3p	Downregulation in HRMECS	Reduce the levels of VEGFA and HIF-*α*, PCNA, and MMPs in cells	Negative regulation in HRMECS	Inhibit the development of DR	[91]
miR-29b-3p	Downregulation in RMECS	miR-29b-3p negatively regulated the expression of angiogenic factors in RMECs	Negative regulation in RMECS	Inhibit the development of DR	[210]
miR-20a	Downregulation in retinal tissues	Upregulation of SIRT1 inhibits the development of DR via miR-20a-induced downregulation of YAP/HIF1*α*/VEGFA	Negative regulation in retinal tissues	Inhibit the development of DR	[154]
miR-152	Downregulation in hRECs and HRMECS	Its overexpression can inhibit VEGF signaling	Negative regulation in hRECs and HRMECS	Inhibit the development of DR	[211]
miR-200b-3p	Downregulation in RMECs	AP1 may exert some promotive effects on the development of DR through its regulation of the MALAT1/miR-200b-3p/VEGFA axis, highlighting that YAP1 silencing may be instrumental for the therapeutic targeting of DR	Negative regulation in RMECs	Inhibit the development of DR	[93]
miR-141–3p	Downregulation in retinal neovascularization and retinal ganglion cells (RGCs)	Impede the activation of the DOK5-mediated MAPK signaling pathway	Negative regulation in retinal neovascularization and retinal ganglion cells (RGCs)	Inhibit the development of DR	[191]
miR-26a	Downregulation in retinal tissues	Reduce the levels of VEGF, IL-1*β*, and NF-*κ*B	Negative regulation in retinal tissues	miR-26a can protect against retinal neuronal impairment in diabetic mice by downregulating PTEN	[182]
miR-145	Downregulation in HRMECS	Reduce the levels of VEGF, IL-1*β*, and NF-*κ*B	Negative regulation in HRMECS	Inhibition of miR-145 abolished the beneficial role of TUG1 knockdown in HG-treated HRMECS. Inhibit the development of DR	[212]
miR-23a	Downregulation in blood and tear	Lack of it increases VEGF expression	Negative regulation in blood and tear	May regulate microvascular growth at the retina via VEGF and contribute to DR progression	[213]
miR-9–3p	Upregulation in Müller glia cells	Mechanistically, exosomal miRNA-9–3p was transferred to retinal endothelial cells and bound to the sphingosine-1-phosphate receptor S1P_1_ coding sequence, which subsequently activated VEGFR2 phosphorylation and internalization in the presence or absence of exogenous VEGF-A	Positive regulation in Müller glia cells	S1P_1_ was identified as the key component of miR-9–3p to regulate abnormal angiogenesis	[171]
miR-1281	Upregulation in serum	miR-1281 positively regulates VEGFA protein expression through activation of VEGFA gene transcription	Positive regulation in serum	Promote the expression of VEGFA	[108]
miR-223–3p	Upregulation in a zebrafish model	Overexpression increases VEGF levels	Positive regulation in a zebrafish model	miR-223–3p negatively regulates eukaryotic translation initiation factor 4E family member 3 (EIF4E3) and insulin-like growth factor 1 receptor (IGF1R)	[214]
miR-181d-5p	Downregulation in HRMECS	miR-181d-5p directly targeted and negatively regulated VEGFA	Negative regulation in HRMECS	miR-181d-5p inhibition augmented cell proliferation, migration, and angiogenesis of HRMECS caused by HG	[215]
miR-139-5p	Upregulation in RMECs	Targeted inhibition PTEN	Positive regulation in RMECs	Promotes cell migration, tube formation, and VEGF protein level	[216]

## Data Availability

Data availability is not applicable to this article as no new data were created or analyzed in this study.
